# Diversity of thermophiles in a Malaysian hot spring determined using 16S rRNA and shotgun metagenome sequencing

**DOI:** 10.3389/fmicb.2015.00177

**Published:** 2015-03-05

**Authors:** Chia Sing Chan, Kok-Gan Chan, Yea-Ling Tay, Yi-Heng Chua, Kian Mau Goh

**Affiliations:** ^1^Faculty of Biosciences and Medical Engineering, Universiti Teknologi MalaysiaSkudai, Malaysia; ^2^Division of Genetics and Molecular Biology, Faculty of Science, Institute of Biological Sciences, University of MalayaKuala Lumpur, Malaysia; ^3^BioEasy Sdn Bhd.Shah Alam, Malaysia

**Keywords:** archaea, biodiversity, culture independent, extremophiles, hot spring, hyperthermophiles, microbial symbiosis, microbiome

## Abstract

The Sungai Klah (SK) hot spring is the second hottest geothermal spring in Malaysia. This hot spring is a shallow, 150-m-long, fast-flowing stream, with temperatures varying from 50 to 110°C and a pH range of 7.0–9.0. Hidden within a wooded area, the SK hot spring is continually fed by plant litter, resulting in a relatively high degree of total organic content (TOC). In this study, a sample taken from the middle of the stream was analyzed at the 16S rRNA V3-V4 region by amplicon metagenome sequencing. Over 35 phyla were detected by analyzing the 16S rRNA data. Firmicutes and Proteobacteria represented approximately 57% of the microbiome. Approximately 70% of the detected thermophiles were strict anaerobes; however, *Hydrogenobacter* spp., obligate chemolithotrophic thermophiles, represented one of the major taxa. Several thermophilic photosynthetic microorganisms and acidothermophiles were also detected. Most of the phyla identified by 16S rRNA were also found using the shotgun metagenome approaches. The carbon, sulfur, and nitrogen metabolism within the SK hot spring community were evaluated by shotgun metagenome sequencing, and the data revealed diversity in terms of metabolic activity and dynamics. This hot spring has a rich diversified phylogenetic community partly due to its natural environment (plant litter, high TOC, and a shallow stream) and geochemical parameters (broad temperature and pH range). It is speculated that symbiotic relationships occur between the members of the community.

## Introduction

Culture-independent techniques enable the comprehensive analysis of microbial populations in hot springs. These approaches involve the use of direct 16S rRNA gene amplification, cloning, and differentiation using denaturing gradient gel electrophoresis (Adrados et al., 2014), terminal restriction fragment length polymorphism analysis (Pervin et al., 2013), or restriction fragment polymorphism analysis (Goh et al., 2011a) before unique clones are sequenced. Next-generation sequencing (NGS) (Thompson et al., 2007) has emerged as a powerful tool, both for elucidating the biodiversity of complex samples and for studying metabolic pathways. The partial 16S-based metagenomics approach (alternatively known as targeted or amplicon metagenomics) has been utilized globally, not only for studying resident microbiota in hot springs (Inskeep et al., 2013), but also for studying coastal waters (Somboonna et al., 2012), soil samples (Fierer et al., 2012), municipal wastewater treatment plants (Cai et al., 2013), tongue-coating microbiomes (Jiang et al., 2012), and the mouse gut (Lee et al., 2010). Many examples of prior studies on the microbial diversity in hot springs are available in the literature, and a few key reports are discussed below. Hot springs adjacent to volcanic environments are often acidic (Urbieta et al., 2014), and the pH is slightly alkaline in areas near limestone. The hot springs at Yellowstone National Park (YNP), USA are one of the most popular sites for thermophile studies (Inskeep et al., 2013), most likely because YNP contains more than 300 geysers with diverse geochemical properties, temperatures, pH conditions, and biological species.

Water pH is an important determinant of microbial diversity in hot springs (Hou et al., 2013). Previously, water samples from an acidic hot spring near the Mutnovsky volcano (70°C, pH 3.5-4) and a circumneutral hot spring from the Uzon Caldera (81°C, pH 7.2-7.4) were analyzed (Wemheuer et al., 2013). Thermotogae and Gammaproteobacteria dominated the Mutnovsky hot spring, while Thermodesulfobacteria, Gammaproteobacteria, and Betaproteobacteria monopolized the Uzon Caldera hot spring. Thaumarchaeota and Crenarchaeota were present in both sites, but Euryarchaeota were only found in the acidic hot spring (Wemheuer et al., 2013). In other reports, the dominant genera found in alkaline hot springs were those of the *Thermus* (De León et al., 2013), *Hydrogenobacter* (Hou et al., 2013), *Caldicellulosiruptor, Dictyoglomus, Fervidobacterium* (Sahm et al., 2013), and *Synechococcus* (Miller and Weltzer, 2011) genera. Among the Archaea, the Crenarchaeal orders *Desulfurococcales* and *Thermoproteales* often predominate in alkaline hot springs (Hou et al., 2013; Sahm et al., 2013).

In addition to pH, water temperatures control microbial distribution within hot springs. In an interesting study, Cole et al. (2013) documented an inversely proportional relationship between hot spring temperatures and the degree of microbial diversity. Thus, temperatures can influence ecosystem compositions. Taxonomic diversity and richness varied along the Bison Pool hot spring outflow channel, wherein a chemotrophic community dominated a biofilm at a high temperature (92°C), while a phototropic mat predominated at a lower temperature (56°C) (Swingley et al., 2012). These findings are in agreement with those from a previous study (De León et al., 2013). In an analyses performed by Vick et al. (2010) using three Little Hot Creek (LHC) hot springs samples, the Aquificae and Thermodesulfobacteria phyla dominated samples from LHC1 (82.5°C, pH 6.75) and LHC3 (79°C, pH 6.97). In contrast, the LCH4 hot spring (78.7°C, pH 6.85) was dominated by the candidate divisions OP1 and OP9, which were first identified in YNP Obsidian Pool (Rohini Kumar and Saravanan, 2010). Water chemistry parameters such as dissolved sulfate, total nitrogen, organic carbon, pyrite, elemental sulfur, and other metal compounds can also influence microbial diversity, as reported by Huang et al. (2013) and Hou et al. (2013).

The Malaysian Sungai Klah (SK) hot spring (located at 3°59′47.88″N, 101°23′35.17″E) is a hotspot for tourism. In this current study, we examined the microbial community of the SK hot spring using a state-of-the-art NGS metagenomics approach. Metagenomes from the Malaysian hot spring water were directly isolated without the need for cultivating microorganisms. The V3-V4 hypervariable regions of prokaryotic 16S rRNA genes were amplified from the metagenome and sequenced directly with an Illumina MiSeq instrument. More than 480,000 sequencing reads of the targeted 16S rRNA genes were generated. In a separate analysis, an additional sample taken from the same site, shotgun metagenomic sequencing data consisting of 552,717,500 reads was generated using an Illumina HiSeq 2500 sequencer to reconstruct the metabolic diversity present in this hot spring.

## Materials and methods

### Water analysis

**Figure 1:**
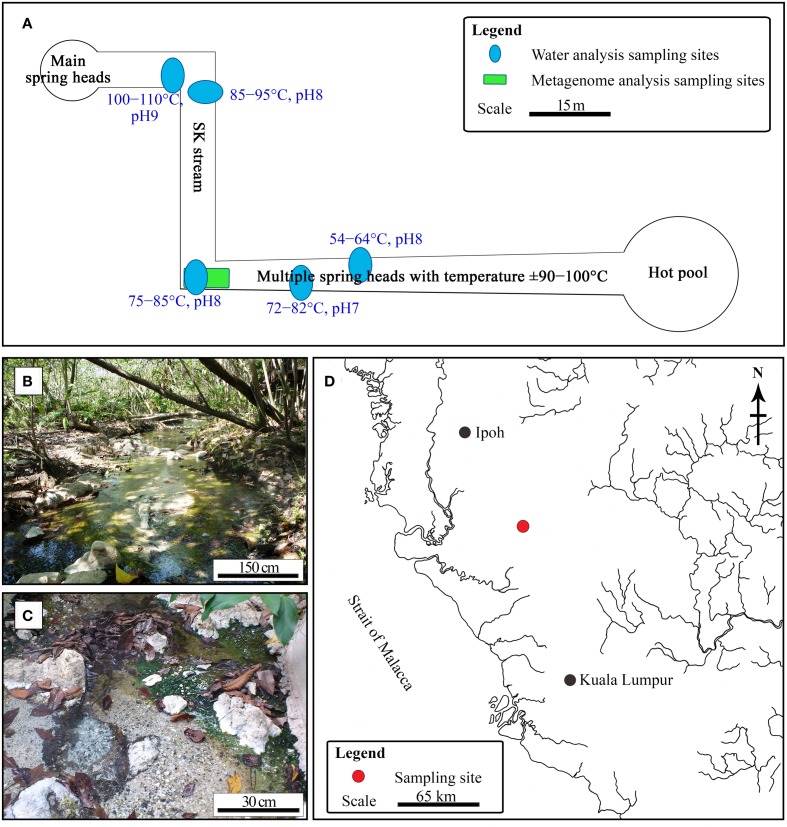
**Sungai Klah hot spring. (A)** Illustration of pH, temperature, and sampling sites for water and metagenomic analyses. **(B,C)** Photographs of the SK hot spring. **(D)** The Sungai Klah hot spring is located in Perak, Malaysia.

The SK hot spring is one of the best-managed recreational hot springs in Malaysia. SK is located near the town of Sungkai and is approximately 130 km from Kuala Lumpur. The SK hot spring is located at a major fault line in Main Range (Titiwangsa Mountain) and is approximately 150 m in length (Figure 1). The Malaysian Main Range is formed by granite, sedimentary rocks, and alkali feldspar (Hussain et al., 2008). Water samples were collected from five locations along the stream of the SK hot spring and mixed in an equal ratio (Figure 1A). The temperature and pH in the stream were measured on-site. The pooled water sample was collected in sterile bottles and stored at 4°C for 2 days prior to physical, chemical, and standard biological analyses. Within a week after sampling, all water analyses (Table S1) were performed by Allied Chemists Laboratory Sdn. Bhd (Malaysia), in accordance with the Public Health Association (APHA) and United States Environmental Protection Agency (USEPA) guidelines.

### Metagenome extraction

A mixture of water and sediment samples was taken from the middle of SK stream (Figure 1A), and the sample was maintained at 4°C for less than a week before analysis. The mixture was shaken vigorously prior to metagenome extraction. A 100-mL sample was centrifuged at 1000 × *g* for 5 min to remove coarse particles, and the water was filtered using a 0.45-μm pore size filter (Sartorius, Göettingen, Germany). The filter membrane was then sliced and subjected to metagenome DNA extraction using the Metagenomic DNA Isolation Kit (Epicentre, Wisconsin, USA), according to the manufacturer's suggested protocol. To increase the purity of the metagenome library, humic acids or other PCR inhibitors were removed using the Agencourt AMPure XP System (Beckman Coulter, Brea, CA, USA). The cleaned metagenome was evaluated by 1% w/v agarose gel electrophoresis, a Nanodrop™ 1000 spectrophotometer (Thermo Scientific, Wilmington, DE, USA), and a Qubit® 2.0 Fluorometer (Invitrogen, Merelbeke, Belgium). Metagenomes extracted from the same sampling site were subsequently analyzed by 16S rRNA sequencing and shotgun metagenome analyses.

### Targeted 16S rRNA fragment library construction, sequencing, and data analysis

Purified metagenomic DNA was used as the template for generating a 16S rRNA metagenome library. The oligonucleotide primers used for this experiment were 5′-TCGTCGGCAGCGTCAGATGTGTATAAGAGACAGCCTACGGGNGGCWGCAG-3′ and 5′-GTCTCGTGGGCTCGGAGATGTGTATAAGAGACAGGACTACHVGGGTATCTAATCC3′, where the underlined regions are the Illumina adapter overhang nucleotide sequences, while the non-underline sequences are locus-specific sequences targeting conserved regions within the V3 and V4 domains of prokaryotic 16S rRNA genes. The locus-specific target sequences were designed based on a reported primer pair, namely S-D-Bact-0341-b-S-17 and S-D-Bact-0785-a-A-21 (Klindworth et al., 2013). The amplified fragments were quantified with the Qubit dsDNA HS Assay Kit (Invitrogen, Merelbeke, Belgium) on a Qubit 2.0 Fluorometer prior to sequencing. Paired-end sequencing of the library was performed on an Illumina MiSeq sequencer (San Diego, CA, USA) using the MiSeq Reagent Kit (v3) with the longest read length set to 2 × 300 base pairs (bp). The resulting sequences were assessed and filtered according to base quality, using the FASTQ Quality Filter (*q* = 20, *p* = 80) of the FASTX-Toolkit. Paired-end reads passing the quality filter were merged using PEAR (Zhang et al., 2014). The successfully merged fragments were searched against the National Center for Biotechnology Information (NCBI) 16S Microbial database using BLASTN (*e*-value ≤10^−6^) of the BLAST+ package (Camacho et al., 2009). The NCBI database was selected because it is a larger database compared to other common databases (i.e., RDP and SILVA) and is therefore capable of providing greater depth of information for archaeal sequences (Kan et al., 2011). Similarity search results were used to analyze the taxonomic distribution of the metagenome sample with MEGAN 5.2.3 (Huson et al., 2011), using the lowest common ancestor (LCA) algorithm (parameter: MinScore = 50, Top Percent = 10, and MinSupport = 5). Rarefaction curves were generated by MEGAN 5.2.3 and are shown in Figure S1. The original sequencing output files have been deposited in the Sequence Read Archive (SRA) service of the European Bioinformatics Institute (EBI) database under Accession Number PRJEB7059.

### Whole metagenome shotgun sequencing and data analysis

The dual-indexed, paired-end library of the metagenome was prepared using the Illumina Nextera DNA Sample Preparation Kit (San Diego, CA, USA), according to the manufacturer's suggested protocols. The metagenome sample library was quantified using a Qubit^®^ 2.0 Fluorometer, and its size distribution was determined using an Agilent 2100 Bioanalyzer (Agilent Technologies, Palo Alto, CA, USA). Whole metagenome shotgun sequencing was performed using the Illumina HiSeq 2500 sequencer (San Diego, CA, USA) available at the High Impact Research Institute at the University of Malaya. For sequencing, we used a dual-indexed 151 (Paired-End sequencing) strategy with a total of 325 cycles (151 bp reads, eight bp index sequence, and seven additional chemistry cycles). The entire sequencing run was completed in approximately 40 h.

Paired-end sequencing reads were filtered with the Trimmomatic 0.30 trimming tool (Bolger et al., 2014) for a minimum terminal base quality score of 20, and only fragments >30 bp were used for generating assemblies. *De novo* assembly of good-quality reads into contiguous sequences (contigs) representing DNA fragments in the metagenome was performed using the IDBA-UD assembler, Version 1.0.9 (Peng et al., 2012). All assembled contigs <300 bp were discarded. Open reading frames (ORFs) in the assembled contigs were predicted using Prodigal gene prediction software, Version 2.60 (Hyatt et al., 2012). Functions of the predicted proteins were based on RAPSEARCH2 2.12 (Zhao et al., 2012) similarity searches against the NCBI GenBank non-redundant protein sequence database (nr) to identify the best hits for each gene. The similarity search results were analyzed using MEGAN 5.2.3 (Huson et al., 2011) by assigning BLAST results to NCBI taxonomies with the LCA algorithm using default parameters. The metagenomic sequences generated in this study were deposited in the EBI SRA under Accession Number PRJEB4990.

## Results

### Water analysis

The temperature along the SK stream ranged between 50 and 110°C, and gas emission by bubbling and visible mist was observed at sites having higher temperatures. The streams have a pH range of 7.0-9.0 (Figure 1A). As it is located within a wooded area, visitors have little access to the stream (Figure 1B).

Water sampling was performed at five different sites along the SK and pooled to obtain representative water samples (Figure 1A). The water quality of the pooled water samples is summarized in Table S1. The mean pH for the SK hot spring was measured at 8.2, with an alkalinity of 76 mg mL^−1^. The following metals were not detected or were below quantifiable limits: cadmium, chromium, copper, lead, manganese, mercury, silver, nickel, and barium. The color of the water was 75 TCU (true color unit), making it significantly higher than the accepted color standard for drinking water (<15 TCU). The high TCU of the SK water is likely due to the presence of organic carbon (i.e., humic acid) and chlorophyll released from the fallen leaves. The SK water met almost all of the quality requirements for drinking water, with the exception of the levels of aluminum, arsenic, and iron, which were above the minimum limits set by the World Health Organization. The presence of these compounds is likely the result of the SK geological setting (Thompson et al., 2007) and is unlikely to be the result of human activity. The SK hot spring contains sulfur, and almost all Malaysian hot springs are rich in sulfur or sulfur compounds. The TOC was 9.04 mg mL^−1^. Using standard bacteriological analysis, coliform and *Escherichia coli* were not detected. The 5-day standard BOD performed at 20°C was measured to be 5 mg L^−1^. As the water temperature of the SK hot spring is high, separate BODs performed at 60 and 80°C yielded values of 10 and 5 mg L^−1^, respectively. Biocides were not detected by gas chromatography-mass spectrometry (National Institute of Standards and Technology reference standards) in the pooled water sample, which was critical for this work to avoid potential biases while studying the natural population of microorganisms in this hot spring.

### Microbial diversity analysis using V3-V4 16S rRNA

**Figure 2:**
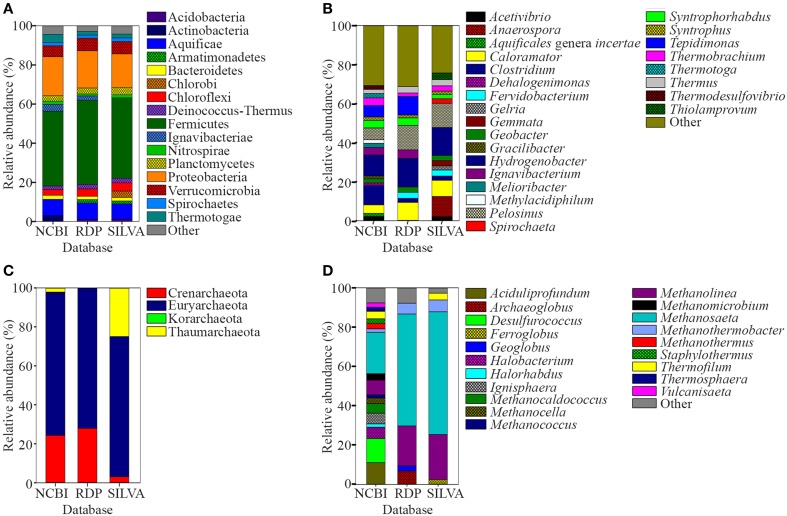
**Relative abundance of prokaryote 16S rRNA sequences classified using NCBI, RDP, and SILVA databases. (A)** Phyla and **(B)** genera levels of prokaryote sequences with relative abundances of <1 and <2%, respectively, were grouped as “Other.” **(C)** Phyla and **(D)** genera levels of archaeal sequences. Genera of archaeal sequences with relative abundances of <2% was grouped as “Other”.

The water and sediment in the middle of the SK stream (Figure 1A) was next analyzed for its microbial biodiversity using primers S-D-Bact-0341-b-S-17/S-D-Bact-0785-a-A-21 (Klindworth et al., 2013). The temperature at this site was 80°C on the sampling day. Using these primers, a total of 480,983 reads was generated by the sequencer. After the quality filtration and sequence read merging process, we obtained 429,677 usable 16S rRNA gene fragments, 97.7% of which were assigned to various taxa. Rarefaction analysis indicated that the sample had reached near saturation for the genus level and higher taxonomic levels, but a curvilinear phase was observed at the species level (Figure S1). A comparison of classification of the archaeal genera to the mothur pipeline against the Ribosomal Database Project (RDP) and SILVA databases is available in Figure 2.

**Figure 3:**
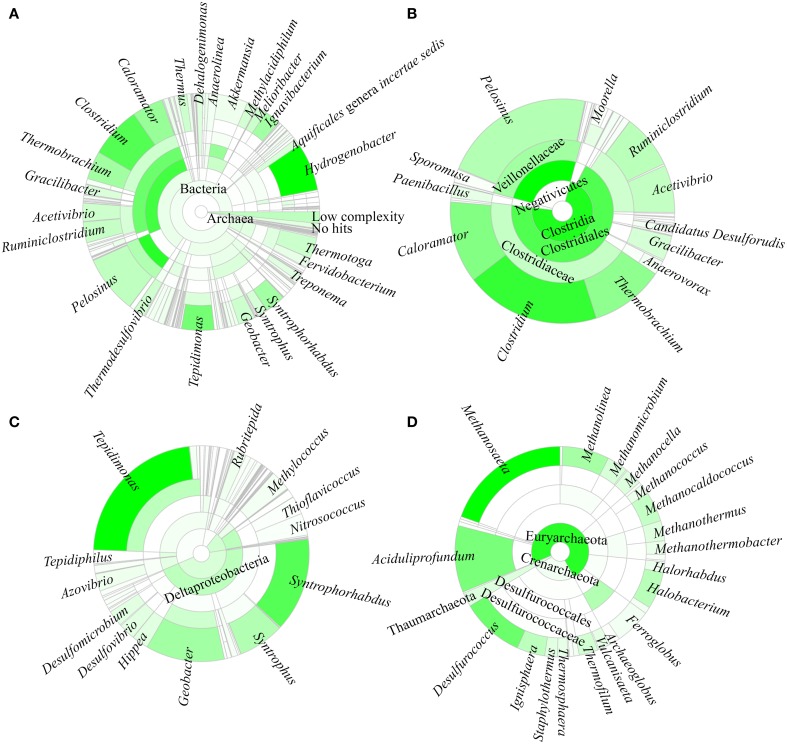
**Taxonomic affiliation of 16S rRNA metagenomic fragments**. The phylogenetic distribution for **(A)** the overall population, **(B)** Firmicutes, **(C)** Proteobacteria, and **(D)** Archaea.

A total of 96.8% of the 16S rRNA gene fragments were assigned to bacteria, whereas 0.91% belonged to Archaea (Figure 3A). These fragments were classified into 67 classes, 120 orders, 206 families, and 358 genera. A total of 35 phyla were present and the major phylum was Firmicutes (37.15%), which comprised mainly of Clostridia and Negativicutes, with Bacilli, Thermolithobacteria, and Erysipelotrichia being present in small proportions (Figure 3B). The majority of the Clostridia were represented by the Clostridiales order. Taxa placed into Clostridiales included generally anaerobic, rod-shaped, typically gram-positive, endospore-forming bacteria and were glycolytic, saccharolytic, peptolytic, and/or chemolithoautotrophic.

As shown in Figure 3C, the second most common phylum was Proteobacteria (19.26% of total 16S rRNA gene fragments). The major class was Deltaproteobacteria (41.96% of the total Proteobacteria), followed by Betaproteobacteria (35.35%), Gammaproteobacteria (11.70%), Alphaproteobacteria (7.56%), and Epsilonproteobacteria (0.32%). The remaining major phyla included Aquificae (8.12% of total fragments), Verrucomicrobia (5.48%), Thermotogae (4.11%), Ignavibacteriae (3.39%), Actinobacteria (2.91%), Chloroflexi (2.90%), Planctomycetes (2.79%), Bacteroidetes (1.97%), Deinococcus-Thermus (1.74%), Nitrospirae (1.56%), Spirochaetes (1.53%), Thermodesulfobacteria (0.82%), Acidobacteria (0.63%), Cyanobacteria (0.62%), and Euryarchaeota (0.58%), while the remaining phyla represented 0.4% of the total population.

*Hydrogenobacter, Clostridium, Pelosinus, Tepidimonas*, and *Caloramator* represented the five major genera (total 36.70% of the entire set of genera) present in the SK hot spring. *Thermobrachium, Syntrophorhabdus, Ignavibacterium, Thermotoga, Melioribacter, Acetivibrio*, and *Geobacter* accounted for approximately 20.31% of the total 16S rRNA gene fragments, whereas another 13.61% of fragments belonged to *Thermus, Thermodesulfovibrio, Methylacidiphilum, Syntrophus, Aquificales genera incertae sedis, Gracilibacter, Dehalogenimonas*, and *Treponema* (Figure 3A). In total, these genera represented approximately 70.62% of the total population within the SK hot spring. Previous studies identified *Anoxybacillus* and *Meiothermus* as the two most common genera isolated from this hot spring (Goh et al., 2011b; Chai et al., 2012); however, these genera represented a small minority of the sequences amplified in the current study. The *Geobacillus* genus, a common thermophile in many hot springs, was also identified as one of the minority genera in this study.

In this study, the Crenarchaeota, Euryarchaeota, Korarchaeota, and Thaumarchaeota phyla were also detected (Figure 3D). Members of the Euryarchaeota phylum accounted for the highest number of fragments, with the leading genera including *Methanosaeta* (30.24% of Euryarchaeota fragments), *Aciduliprofundum* (15.93%), *Methanolinea* (10.68%), *Halobacterium* (8.27%), *Methanocaldococcus* (7.04%), *Methanomicrobium* (4.75%), *Methanocella* (4.02%), and *Methanothermus* (3.91%). To date, only four species of Euryarchaeota have been described in the *Methanosaeta* genus, including *Methanosaeta concilii* (Patel and Sprott, 1990), *Methanosaeta thermophila* (Kamagata et al., 1992), *Methanosaeta harundinacea* (Ma et al., 2006), and *Methanosaeta pelagica* (Mori et al., 2012), with *Methanosaeta thermophila* exhibiting the highest growth temperature (55-60°C). In relative terms, 16S rRNA gene fragments closest to *Methanosaeta thermophila* were most commonly identified in SK, among the *Methanosaeta* genus.

Aquificae and Thermotogae were the only two known hyperthermophilic bacterial phyla that exhibit high heat tolerance. In this work, most Aquificae genera detected were *Hydrogenobacter* or *Aquificales* genera *incertae sedis* (Latin: uncertain placement), contributing more than 92% of the total Aquificae, whereas the remaining small percentage were represented by *Thermovibrio, Persephonella*, and *Sulfurihydrogenibium*. The detected *Hydrogenobacter* 16S rRNA gene fragments showed the closest similarity to *Hydrogenobacter subterraneus*, which was first isolated from a Japanese deep subsurface geothermal water pool (Takai et al., 2001) and is described as a strictly aerobic heterotroph with optimum growth at 78°C and pH 7.5 (close to the temperature and pH of the SK sampling site). In relative terms, the number of Thermotogae detected was less than that of Aquificae. The gene fragments putatively assigned to *Thermotoga thermarum* and *Thermotoga neapolitana* corresponded to 90.91% of the total Thermotogae, whereas the *Fervidobacterium*, and *Thermosipho* genera represented the minority populations. At present, the genus *Thermotoga* consists of nine species, at least three of which were potentially present in SK hot spring.

Several thermophilic photosynthetic microorganisms were detected in the SK hot spring. These microorganisms included members of the *Roseiflexus, Porphyrobacter*, and *Chloroflexus* genera. The *Chloroflexus* is a filamentous anoxygenic phototrophic bacterium. *Chloroflexus aggregans* can grow phototrophically under anaerobic conditions, but also exhibits the ability to grow chemotrophically under aerobic and dark conditions. The complete *Chloroflexus aggregans* genome was published previously (Tang et al., 2011). In addition, several photosynthetic cyanobacteria, including bacteria from the *Cyanobacterium, Synechococcus, Gloeobacter*, and *Oscillatoria* genera were also detected in the SK hot spring. “*Candidatus Chloracidobacterium thermophilum*,” a chlorophyll-based photoheterotroph, was also present.

The SK hot spring is slightly alkaline. Interestingly, several acidophiles were present within the community. For an example, ~1% of 16S rRNA gene fragments was related to *Methylacidiphilum* (of the Verrucomicrobia phylum). The optimum growth condition for *Methylacidiphilum infernorum* is pH 2-2.5, and the optimal growth temperature is 60°C (Hou et al., 2008). Other thermoacidophiles present included species closest to *Acidimicrobium ferrooxidans*, which growth optimally at pH 2 (Clum et al., 2009), *Syntrophus aciditrophicus* (pH 5-6.2) (Kulichevskaya et al., 2008), *Acidisphaera rubrifaciens* (pH 4.5-5) (Hiraishi et al., 2000), *Acidothermus cellulolyticus* (pH 5) (Mohagheghi et al., 1986), euryarchaeotal *Aciduliprofundum boonei* (pH 3.3-5.8) (Reysenbach et al., 2006), *Acidithiobacillus caldus* (pH 2) (Kelly and Wood, 2000), and *Sulfolobus acidocaldarius* (pH 2) (Chen et al., 2005). We spread the collected sample on acidic medium (pH 4.0), and thermoacidophiles colonies were able to form on the plates (data not shown).

In addition to “*Ca*. *Chloracidobacterium thermophilum*,” as described above, we also detected the presence of other uncultivable *Candidatus*, including “*Ca*. Solibacter usitatus,” “*Ca*. Koribacter versatilis,” “*Ca*. Desulforudis audaxviator,” “*Ca*. Midichloria mitochondrii,” “*Ca*. Accumulibacter phosphatis,” “*Ca*. Cloacimonas acidaminovorans,” and “*Ca*. Korarchaeum cryptofilum.” Little is known about these microorganisms, and it is exciting to document their presence in the SK hot spring, although their functional contributions to microbiome formation are presently unknown. In addition to the microorganisms mentioned here, certain fragments revealed no close relatives in the current NCBI database, apparently indicating the presence of novel organisms indigenous to the SK hot spring.

### Whole genome shotgun metagenome analysis

The sampling site for shotgun analysis was identical to the one used for the 16S rRNA metagenome sequencing (Figure 1A). Detailed information regarding the sequencing reads and assembled results are summarized in Table 1. A total of 552,717,500 reads was generated from the HiSeq 2500 sequencer. These reads were trimmed and assembled into more than 900,000 contigs. MEGAN software was primarily used to display and analyze the data. The majority of the contigs had relatively good coverage. The average coverage was 28.7X, and the total number of contigs with greater coverage than the average coverage value was 133,616. A total of 278,434 contigs had coverage exceeding 10X.

**Table 1:** **HiSeq sequencing data**.

**Characteristics**	**Amount**
**SEQUENCING STATISTICS**	
Number of raw reads generated	552,717,500
Raw bases generated	82,907,625,000
**ASSEMBLY STATISTICS**	
Number of assembled contigs	922,897
Largest contig size	404,091
N50 contig size	2331
Total # large contigs (>300 bp)	694,949
Total # bases large contigs	1,231,473,681 bp
Average large contig size	1772 bp
N50 large contig size	2693 bp
Total assembled contig length	1,324,524,733 bp
Total # unassembled reads	168,538,065

Overall, 88.44% of the predicted ORFs (457,296 proteins) belonged to bacteria, and 10.14% (52,408 proteins) and 0.67% (3446 proteins) were from Archaea and Eukaryota, respectively. A small fraction of the total contigs belonged to viruses and unclassified sequences. A total of 83 phyla were identified, and the top 20 phyla included (in decreasing order of prevalence) Firmicutes, Proteobacteria, Chloroflexi, Bacteroidetes, Euryarchaeota, Crenarchaeota, Deinococcus-Thermus, Spirochaetes, Nitrospirae, Planctomycetes, Aquificae, Ignavibacteriae, Acidobacteria, Thermotogae, Cyanobacteria, Dictyoglomi, Actinobacteria, Thermodesulfobacteria, Thaumarchaeota, and the candidate division OP1.

Approximately 7.11% of the ORFs were assigned to *Clostridium*. Other genera with a large number of ORFs included *Anaerolinea* (3.39%), *Acetivibrio* (3.35%), *Thermodesulfovibrio* (2.58%), *Ignavibacterium* (2.25%), and *Meiothermus* (2.18%). The above six genera accounted for 20.85% of the total ORFs found in this study. The genera of *Caldilinea* (1.58%), *Dictyoglomus* (1.39%), *Gemmata* (1.37%), *Chloroflexus* (1.31%), *Treponema* (1.30%), *Archaeoglobus* (1.26%), *Geobacter* (1.17%), *Roseiflexus* (1.06%), *Paenibacillus* (1.02%), and candidate division OP1 (0.98%) contributed an additional 12.45% to the total set of ORFs identified.

Phylum assignments for the identified ORFs were compared with the 16S rRNA metagenomic data. In general, the distribution of the major phyla was similar between both the 16S rRNA and shotgun metagenome approaches. Nevertheless, three phyla were detected in 16S rRNA diversity study, but not by the shotgun sequencing method. These phyla include Haloplasmatales, Lentisphaerae, and Armatimonadetes (also known as candidate phylum OP10). However, the primers used to amplify metagenomes targeting the 16S rRNA V3-V4 region missed phyla of the candidate divisions OP1 and JS1, environmental bacteria, some Archaea samples [Fusobacteria, Poribacteria, Chrysiogenetes, Fibrobacteres, Nanoarchaeota, Candidatus Saccharibacteria (formerly known as candidate division TM7)], and candidate division WS3 (Latescibacteria). However, some of these phyla are mesophilic and it is thus likely that they may have originated from the soil. Using the shotgun metagenome approach, gene fragments of viruses were detected, and negligible numbers of fungi, insect, algae, protozoa, and parasites protists were identified as well.

### Microbial functional gene diversity

**Figure 4:**
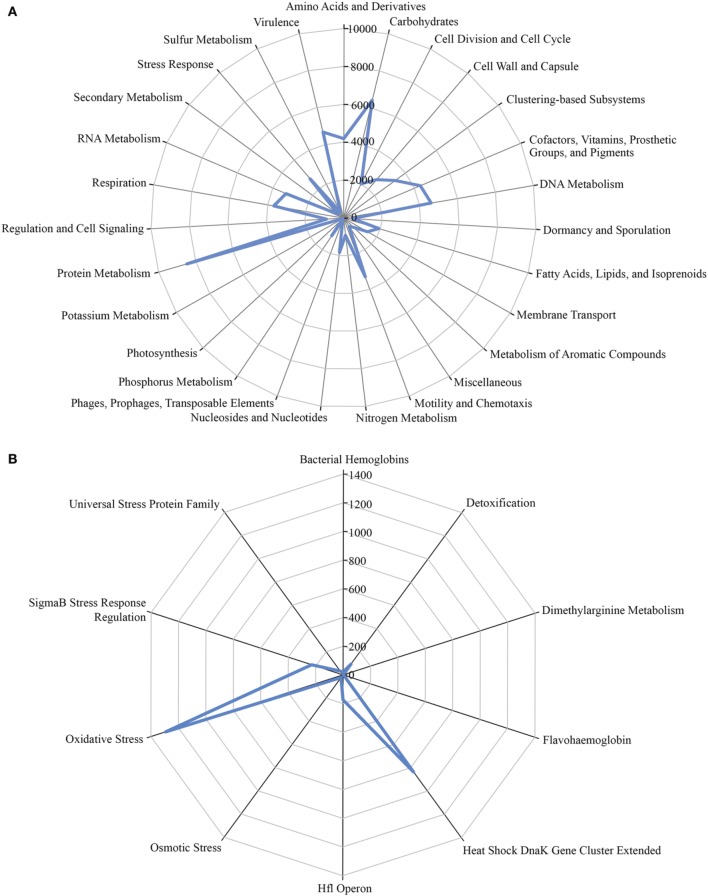
**The number of predicted ORFs that matched metabolic categories, based on SEED subsystem. (A)** Major metabolic categories. **(B)** Stress responses.

**Figure 5:**
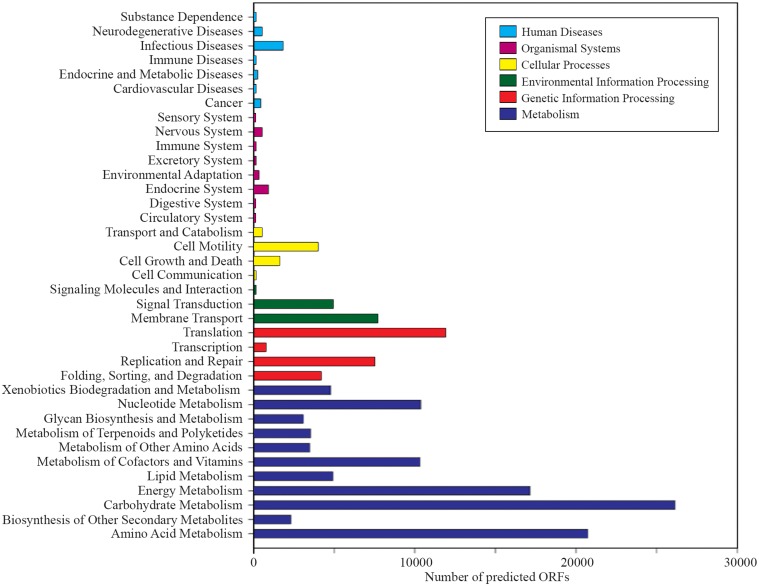
**Number of predicted ORFs that matched metabolic categories, based on KEGG analysis**.

A total of 1,203,458 full-length protein-coding genes identified within the shotgun metagenome dataset were analyzed. Among these, 817,831 ORFs were annotated based on the closest match in the GenBank NR protein database. Using the SEED and KEGG function of MEGAN, the ORFs were classified according to their putative functions (Figures 4, 5). We used these sequence affiliations to further understand the relationship between the geochemical parameters and the population diversity within the SK hot spring.

#### Carbon metabolism function within the SK hot spring community

**Figure 6:**
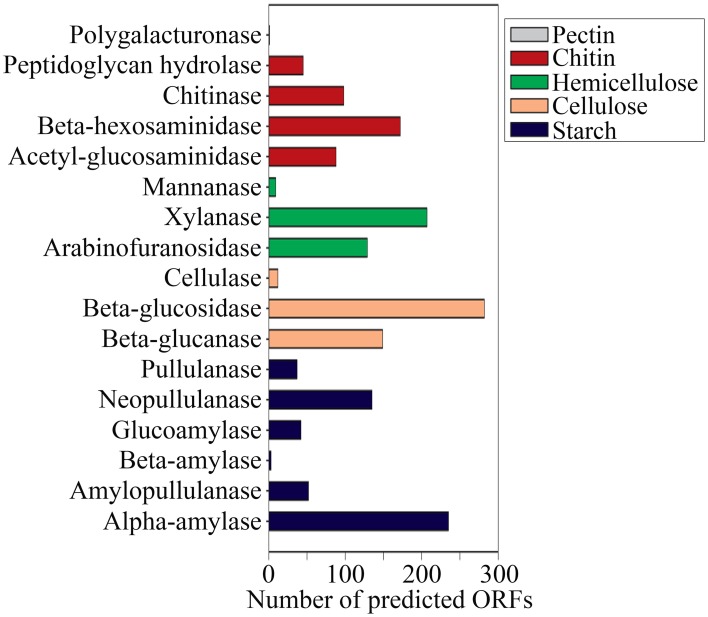
**Gene mining for enzymes involved in carbon (polysaccharides) degradation**.

The reactions involved in carbon metabolism include carbon degradation, carbon fixation, and methane metabolism. Carbon degradation enzymes were identified using dbCAN CAZy (Yin et al., 2012), and their presence is illustrated in Figure 6. These enzymes include genes encoding alpha-amylase, amylopullulanase, beta-amylase, glucoamylase, neopullulanase, and pullulanase (involved in starch degradation); beta-glucanase, beta-glucosidase, and cellulase (involved in cellulose degradation); arabinofuranosidase, xylanase, and mannanase (involved in hemicellulose degradation); acetyl-glucosaminidase, beta-hexosaminidase, chitinase, and peptidoglycan hydrolase (involved in chitin degradation); polygalacturonase (involved in pectin degradation); and other carbohydrate degradation enzymes. Because the SK hot spring is physically located within a woodland, the stream is often fed by fallen leaves, twigs, branches, and even tree trunks. Thus, the SK microbial community is likely able to acquire carbon sources from fallen plants and other natural organic matters. Furthermore, the absence of carbon starvation stress-related proteins in the shotgun metagenome data (Figure 4B) suggested that the TOC content in the SK hot spring might be sufficient for the survival of the community.

**Figure 7:**
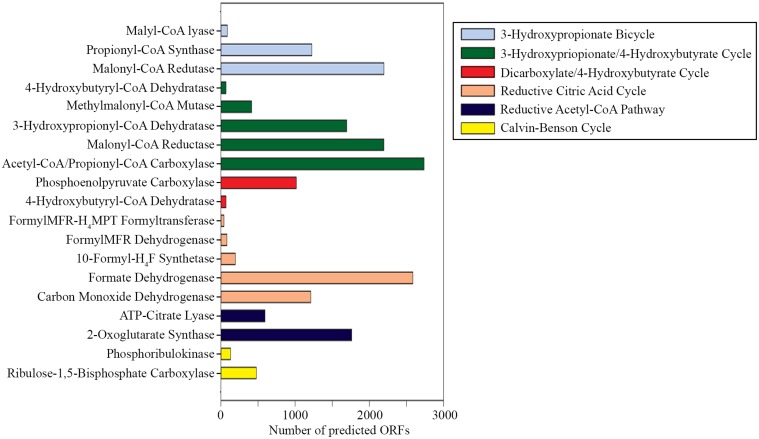
**Gene mining for the key enzymes involved in autotrophic carbon fixation**. (Abbreviations: FormylMFR-H4MPT formyltransferase, formylmethanofuran-tetrahydromethanopterin formyltransferase; 10-formyl-H4F synthetase, 10-formyl-tetrahydrofolate synthetase).

**Figure 8:**
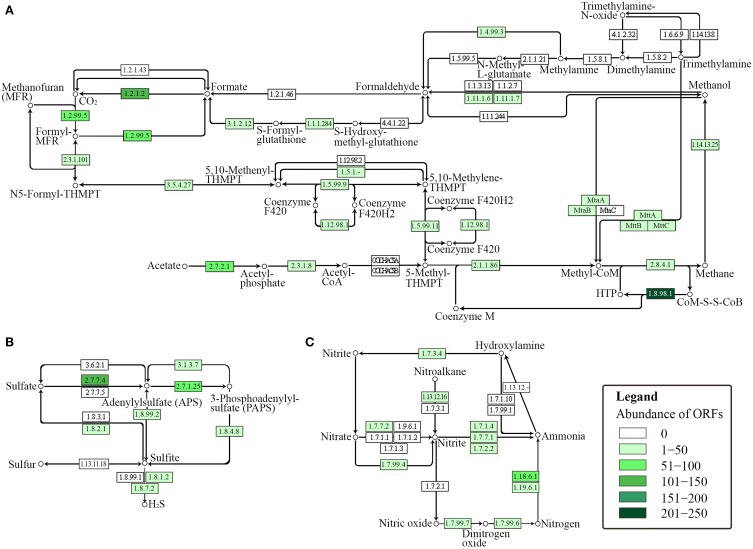
**KEGG-based functional analysis of the SK hot spring community**. Each numbered rectangle represents participating enzymes. The boxes are shaded according to the number of ORFs assigned to the corresponding function in each dataset: **(A)** methane metabolism; **(B)** sulfur metabolism, and **(C)** nitrogen metabolism.

The presence of key enzymes involved in six autotrophic carbon fixation pathways (Figure 7) was associated with the presence of (i) cyanobacteria and the α-, β-, and γ-subdivisions of Proteobacteria for the Calvin-Benson cycle; (ii) green sulfur bacteria (Chlorobi), Aquificae, Nitrospira, and ε-Proteobacteria for the reductive tricarboxylic acid cycle; (iii) acetogenic Firmicutes, anammox Planctomycetes, and methanogenic Euryarchaeota for the reductive acetyl-CoA pathway; (iv) anaerobic and microaerobic autotrophic Thermoproteales and Desulfurococcales for the bicarboxylate/4-hydroxybutyrate cycle; (v) aerobic autotrophic Sulfolobales for the 3-hydroxypropionate/4-hydroxybutyrate cycle; and (vi) phototrophic green non-sulfur bacteria of the family Chloroflexaceae for the 3-hydroxypropionate bicycle. Shotgun metagenome sequencing also detected enzymes involved in four types of methanogenic pathways (Figure 8A) utilizing carbon dioxide, methanol, acetate, and methylamine for methane production, as well as methane monooxygenase and formate dehydrogenase for methane consumption. Methanogenic thermophiles were therefore confirmed to be present in SK hot spring. The 16S rRNA survey also identified the presence of methanotrophs, including the Euryarchaeota.

#### Sulfur metabolism within the SK hot spring community

In general, sulfur metabolism involves sulfur oxidation and sulfur reduction. Because most of the ORFs detected in shotgun metagenome data were related to the sulfate reduction pathway, we conclude that the SK community preferably generates the reductive form of sulfur compounds (Figure 8B). The pathway involves the conversion of sulfate into adenylylsulfate and the further generation of 3′-phosphoadenylylsulfate, sulfite, and hydrogen sulfide (H_2_S). The generation of hydrogen sulfide is an important transformation in sulfur metabolism because it can be used for the biosynthesis of sulfur-containing amino acids. However, the absence of homocysteine desulfhydrase (EC 4.4.1.2) suggests that methionine may be synthesized through another pathway. With respect to the sulfur oxidation pathway, although a gene for sulfur dehydrogenase was detected, the SK shotgun metagenome data did not reveal sulfur dioxygenase or sulfite oxidase ORFs.

Sulfate-reducing microorganisms are important in degrading organic matter under anoxic environments. In the SK community, the organisms related to sulfate reduction included those having closest ORFs hits to *Thermodesulfovibrio yellowstonii* (Henry et al., 1994), *Thermodesulfovibrio aggregans* (Sekiguchi et al., 2008), *Desulfomicrobium thermophilum* (Thevenieau et al., 2007), *Desulfotomaculum carboxydivorans* (Parshina et al., 2005), *Desulfotomaculum kuznetsovii* (Visser et al., 2013), *Thermodesulfatator indicus* (Moussard et al., 2004), *Thermodesulfobacterium commune* (Zeikus et al., 1983), *Thermodesulfobium narugense* (Mori et al., 2003), *Archaeoglobus veneficus* (Huber et al., 1997), and *Caldivirga maquilingensis* (Itoh et al., 1999). Sulfur-reducing microorganisms use sulfur for the generation of hydrogen sulfide because they are unable to reduce sulfate. Examples of sulfur-reducing microorganisms in the SK community include *Hippea maritima* (Miroshnichenko et al., 1999), *Thermococcus gammatolerans* (Jolivet et al., 2003), *Thermofilum pendens* (Anderson et al., 2008), *Caldivirga maquilingensis* (Itoh et al., 1999), *Vulcanisaeta distributa* (Itoh et al., 2002), and *Vulcanisaeta moutnovskia* (Prokofeva et al., 2005; Gumerov et al., 2011).

#### Nitrogen metabolism within the SK hot spring community

The nitrogen cycle is a complex biological process that requires interplay among many microorganisms in catalyzing different reactions. The annotation of sequences relevant to nitrogen metabolism in the shotgun metagenome revealed the presence of genes involved with complex nitrogen immobilization and the mineralization cycle (Figure 8C). The presence of nitrogenase genes involved in nitrogen fixation having the closest hits to *Thermodesulfovibrio* spp., *Roseiflexus* spp., *Methanosaeta* spp., and *Clostridium* spp. (Chen, 2005) indicated the potential of the SK hot spring community for fixing atmospheric nitrogen to ammonia. Genes related to both assimilatory and dissimilatory nitrate reduction pathways were also detected. This finding indicates the existence of distinct mechanisms for the transformation of nitrate into nitrite, nitric oxide, dinitrogen oxide, ammonia, and nitrogen in the SK community. Due to the low oxygen content of the SK hot spring, it is not surprising to detect the presence of denitrifying bacteria that are able to reduce nitrate or nitrite as terminal electron acceptors for respiration. However, thermophilic denitrifying bacteria were not detected in 16S rRNA metagenome analysis; instead the population was dominated by mesophiles. This shows that most of the thermophiles in SK are unlikely to undergo denitrification processes, and such genes plausibly originated from mesophilic populations located in upper stream regions with a lower temperature.

### Evaluation of the presence of pathogenic strains in hot springs

A total of 4659 and 2818 ORFs were related to various virulence factors and pathogens associated with human diseases, respectively. It is not uncommon to observe the presence of viruses or phages in hot springs. For example, viruses and phages were also detected in the Bear Paw and Octopus hot springs in YNP (Pride and Schoenfeld, 2008). Most of the viruses identified in the present study belonged to the Caudovirales order. The Lipothrixviridae family viruses that infect Archaea were present in lesser quantities. We also detected some Phycodnaviridae viruses, which infect marine or freshwater eukaryotic algae. Besides, as evident from the 16S rRNA NGS data, pathogens detrimental to the health of humans or animals identified included those shown to have the closest hits to *Clostridium difficile* (total 16S rRNA fragments: 234), *Clostridium hiranonis* (7), *Brucella suis* (14), *Legionella pneumophila* (29), *Leptospira licerasiae* (267), *Leptospira wolffii* (12), *Pseudomonas fluorescens* (18), *Rickettsia montanensis* (10), *Rickettsiales* genera *incertae sedis* (170), and others. Genome fragments from the above pathogens were also detected in the shotgun metagenome. In addition, fragments of genomes of some other pathogenic strains were detected in the shotgun metagenome, but were not detected in the 16S rRNA analysis, including *Burkholderia, Campylobacter, Cryptosporidium, Enterococcus, Escherichia, Giardi, Legionella, Leptospira, Mycobacterium, Salmonella, Shigella, Vibrio*, and *Yersinia* spp.

## Discussion

Environmental samples represent an enormous reservoir of genetic diversity from archaea, bacteria, eukaryotes, and viruses. Approximately 99% of microorganisms are not amenable to cultivation by standard laboratory techniques. Metagenome sequencing is a powerful approach in studying the environmental genetic diversity directly by bypassing the limitation of cultivation-based method (Sharon and Banfield, 2013). Metagenomics sequencing is achieved through two approaches: amplicon sequencing and shotgun sequencing. In this current work, primers S-D-Bact-0341-b-S-17/S-D-Bact-0785-a-A-21 pair targeting the V3-V4 region of prokaryotic 16S rRNAs (Klindworth et al., 2013) was used. Based on the analysis, *in silico* evaluation for more than 370,000 sequences, these primers enable overall coverage of approximately 94.5% bacterial and 64.5% archaeal sequences in the database searched if one *in silico* nucleotide mismatch is considered (Klindworth et al., 2013).

Most of Malaysian hot springs appear as a pool or basin where the water is stagnant or experiences little exchange. Trees grow along the banks of the SK hot spring. The SK hot spring is richer in aluminum, iron, sulfate, and sulfur in comparison to other Malaysian hot springs, such as the Semenyih, Kampung Serai, and IKBN Hulu Langat hot springs. For example, the total nitrogen content detected in the SK hot spring was twice that of the Kampung Serai and IKBN Hulu Langat hot springs. When compared to 60 other Malaysian hot springs, the SK hot spring is unique due to the natural environment of the site. For example, (i) the ~150-m-long hot spring contain multiple spring heads with temperatures exceeding >100°C. As the stream is shallow, (ii) the temperature along the streams fluctuate with a range of 50 to 110°C. (iii) The pH along the streams is not uniform and ranges between values of 7.0 and 9.0, and (iv) the SK hot spring is fed with plant litters (Figures 1B,C). The plant litter is one of the natural sources that enhance its carbon contents. The TOC for the SK hot spring was determined as 9.04 mg mL^−1^, while less than 1 mg mL^−1^ TOC was measured in the Semenyih, Kampung Serai, and Hulu Langat hot springs where plant litter is absent (unreported). It is possible that plant litter enriches the SK microbiome diversity of thermophiles by providing additional carbon sources where the emerging ground water is lacking. It is also possible that combinations of the four aforementioned factors (i-iv) increase the biodiversity of the SK hot spring.

Previously, Vick et al. (2010) used 16S rRNA gene libraries to analyze the microbial diversity present in the Little Hot Creek (LHC) hot springs located near to California Mammoth Lakes. Water emerging from the ground of LHC flows as a stream at a temperature (78.7-82.5°C) and pH (6.75-6.97) similar to that of SK. The dominant phyla in LHC are Aquificae, Thermodesulfobacteria, Deinococcus–Thermus, Thermotogae, Chloroflexi, and Dictyoglomi. This contrasts with SK, where approximately 56% of the phyla were composed of Firmicutes and Proteobacteria. In one biodiversity analysis, the population of archaeal and bacterial communities taken over 3 years from three alkaline hot springs in the Heart Lake Geyser Basin (HLGB) at YNP was analyzed (De León et al., 2013). One of the HLGB hot springs is a 2-m wide, basin-type hot spring. Its temperature and pH (75°C, pH 8.5) were relatively constant over the 3-year sampling period, and the bacterial populations identified (seven major bacterial phyla) were stable over time. Yet, Crenarchaeota and Thaumarchaeota dominated this site and the archaeal phyla composition changed over time. In this study, we observed that the SK hot spring exhibits greater biodiversity than present in the HLGB. Cole et al. (2013) proposed that temperature can control the diversity of hot spring microbial communities. Using alpha diversity analysis tools, the number of Operational Taxonomic Unit at higher temperatures was significantly lower than that at lower temperatures. Relative to previous findings by Vick et al. (2010), Swingley et al. (2012), Hou et al. (2013), Cole et al. (2013), and De León et al. (2013), the microbial diversity within the SK hot spring is comparatively distinct and richer in biodiversity, which plausibly stems from a combination of the four factors described above.

Dissolved oxygen levels are inversely proportional to water temperatures. Given that the water temperature at the sampling site was high (80°C), the dissolved oxygen within SK hot spring was expected to be low. Based on the 16S rRNA sequencing, the majority of the population (approximately 70%) was comprised of strict anaerobes, whereas the remaining microbes were either facultative or aerobic. Surprisingly, one of the dominant thermophile populations identified was *Hydrogenobacter* spp., which are obligate chemolithotrophic organisms. The 16S rRNA fragments of *Hydrogenobacter* spp. represented approximately 5% of the total population, which was two-fold higher than the next most abundant anaerobic taxa. Question that naturally arises are why aerobic *Hydrogenobacter* species would dominate the community, and how other aerobic thermophiles survived in an anoxic environment. Below are two possible answers to these questions. First, the SK hot spring is shallow, and the water is continuously aerated by the movement of the current. Second, the presence of photosynthetic bacteria likely supply additional dissolved oxygen. Several spots of green biomats are present along the SK hot spring with temperatures ranging between 50 and 70°C. Although the diversity of microorganisms that form the biomat have yet to be determined, it is believed that the mat is composed of the photoautotrophic bacteria mentioned earlier.

From the analysis of carbon metabolism, the SK hot spring community uses diverse means for growth. The thermophiles can use organic or inorganic substrates. A fraction of the community exhibits a complete metabolic pathway, whereas the others may benefit from syntrophic relationships. An example of such a syntrophic interaction is found among acetogenic bacteria and methanogenic Archaea with respect to the methanogenesis process (Ragsdale and Pierce, 2008). Another possible partnership may occur between *Treponema caldaria* and *Clostridium thermocellum* for the enhancement of cellulose degradation (Pohlschroeder et al., 1994). It is also not surprising that certain microorganisms in the SK community contain more than one carbon fixation pathway that utilize different inorganic carbon species, as found in *Roseiflexus* spp. (Schaffert et al., 2012).

Another example of symbiotic relationships is observed between the photosynthetic “*Ca. Chloracidobacterium thermophilum*” with *Anoxybacillus* spp. (facultative) and *Meiothermus* spp. (aerobic) (Garcia Costas et al., 2012). The genome of photosynthetic “*Candidatus Chloracidobacterium thermophilum*” has been sequenced (Garcia Costas et al., 2012), but the bacteria is very difficult to cultivate in the laboratory. Thus far, this *Candidatus* can only be co-cultured in a mixture of *Anoxybacillus* and *Meiothermus* spp., and the latter two genera were also present in the SK hot spring. The “*Ca*. *C. thermophilum*” lacks sulfate reduction function and is complemented by *Anoxybacillus* and *Meiothermus*. In addition, the presence of phototrophic microorganisms helps to increase the level of dissolved oxygen in the heated water that is needed by the other aerobic strains.

The community appears to survive using mutualistic or commensalistic symbiotic relationships to thrive under multiple environmental stresses. Together, the uniqueness of the diversified pathways observed is likely a result of the physical characteristics of the hot spring and additional factors, such as dissolved gases, minerals, and trace elements. For the metabolism of nitrogen and sulfur, certain key enzymes (i.e., nitrite reductase, ammonia monooxygenase, sulfite oxidase, and sulfur dioxygenase) were not identified in this work. The absence of certain ORFs in the pathway may not indicate the absence of such functions; instead, this missing information may be due to low gene abundance or missed detection during metagenome sequencing. Furthermore, all of these pathways are greatly influenced by the environmental conditions, such as temperature, oxygen, light, organic matter content, and their availability (Shapleigh, 2009).

Generally, the non-scientific public tends to believe that geothermal springs are safe, whereas biologists appreciate that these sites are natural growth bioreactors for thermophiles. Fragments of genomes and 16S rRNA sequences of several pathogens such as amoebas or viruses, and other mesophiles were found in this study, which raises concerns for public safety. Nevertheless, the SK hot spring was not dominated by these pathogens, as the numbers of total fragments were not significant when compared to microbiome as a whole. Therefore, SK hot spring is generally safe for the public. It remains to be determined why mesophilic pathogens were present in the SK hot spring with temperatures of approximately 80°C. We are unable to rule out the possibility that the original source of these cells may be the soil or other objects that were introduced into the hot spring, as we also detected the gene fragments of Basidiomycota (mushroom), Nematoda, and Platyhelminthes (worms), and it is unlikely that these organisms can live at 80°C.

### Conflict of interest statement

The authors declare that the research was conducted in the absence of any commercial or financial relationships that could be construed as a potential conflict of interest.

## Supplementary material

Figure S1 **Rarefaction curves created with MEGAN software**.
