# Supplementary figures and images for: Diversity of thermophiles in a Malaysian hot spring determined using 16S rRNA and shotgun metagenome sequencing
Source: Front Microbiol. 2015 Mar 5;6:177. doi: 10.3389/fmicb.2015.00177 (PMC4350410; doi:10.3389/fmicb.2015.00177)

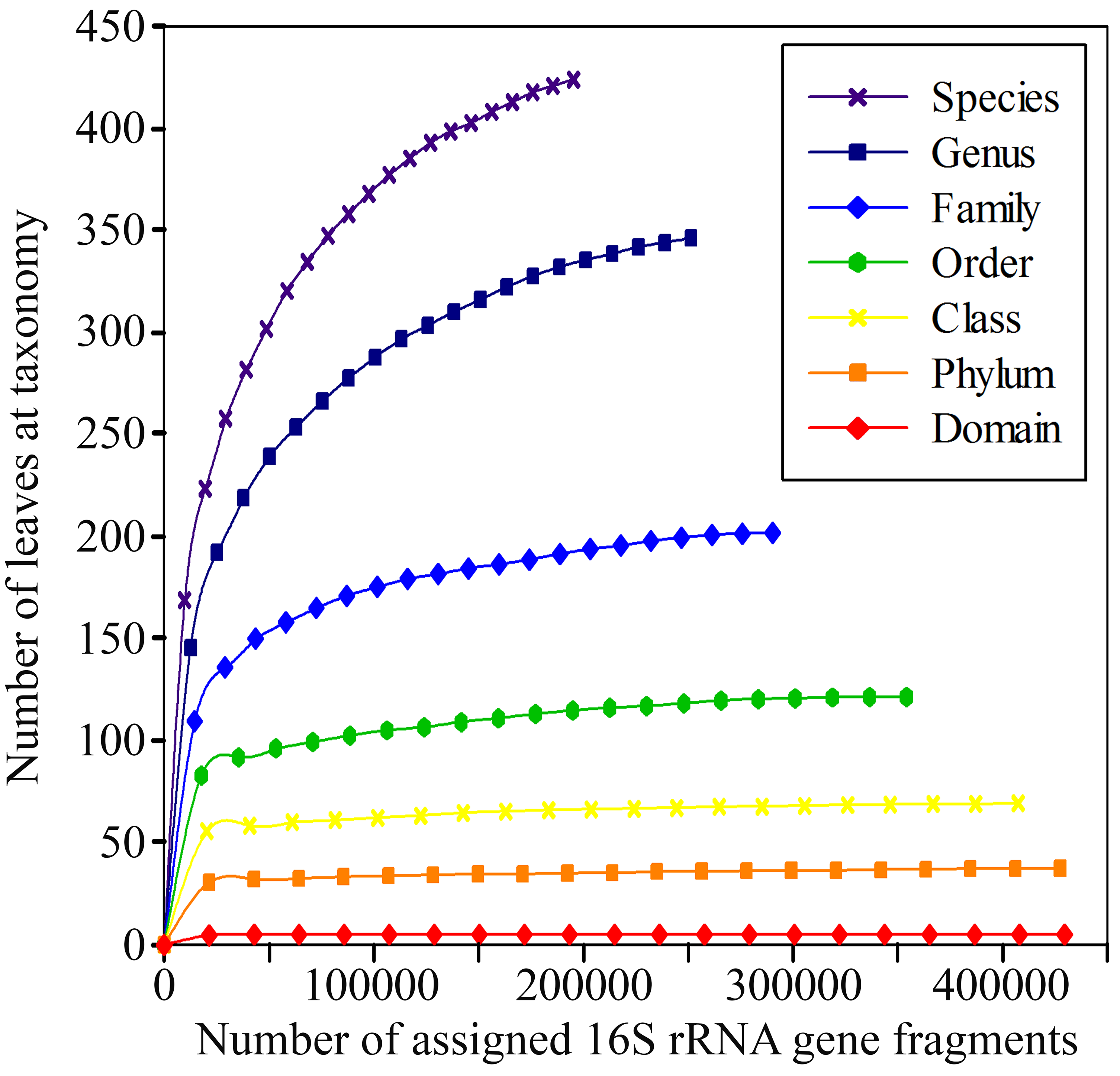

Supplement: Figure S1 — Rarefaction curves created with MEGAN software. [file Image1.TIF]
