# Supplementary material for: Diversity of thermophiles in a Malaysian hot spring determined using 16S rRNA and shotgun metagenome sequencing
Source: Front Microbiol. 2015 Mar 5;6:177. doi: 10.3389/fmicb.2015.00177 (PMC4350410; doi:10.3389/fmicb.2015.00177)
Supplement: Supplementary file 2 [file Table1.DOCX]

**Table S1 |** Water analysis of the pooled Sungai Klah hot spring sample.

| **Test parameter** | **Method** | **Unit** | **Results** | **WHO** |
| --- | --- | --- | --- | --- |
| **Physical** |  |  |  |  |
| Color | APHA 2120 B | TCU | 75 | 15 |
| Turbidity | APHA 2130 B | NTU | 130 | 5 |
| **Chemical** |  |  |  |  |
| pH | APHA 4500 H^+^ B | - | 8.2 | 6.5−8.5 |
| Aluminium (Al) | APHA 3030 F / USEPA 6010 B | mg L^-1^ | 0.96 | 0.2 |
| Ammonia (N) | APHA 4500-NH_3_-C | mg L^-1^ | ND (< 0.1) | 1.5 |
| Ammoniacal nitrogen | APHA 3030 F / USEPA 6010 B | mg L^-1^ | < 0.2 | - |
| Anionic Detergent (MBAS) | APHA 5540 C | mg L^-1^ | ND (< 0.2) | - |
| Arsenic (As) | APHA 3030 F / USEPA 6010 B | mg L^-1^ | 0.07 | 0.01 |
| Barium (Ba) | APHA 3030 F / USEPA 6010 B | mg L^-1^ | ND (< 0.02) | 0.7 |
| Boron (B) | APHA 3030 F / USEPA 6010 B | mg L^-1^ | 0.06 | 2.4 |
| Biocides^a^ | GC - In House^b^ | mg L^-1^ | ND | - |
| Cadmium (Cd) | APHA 3030 F / USEPA 6010 B | mg L^-1^ | ND (< 0.002) | 0.003 |
| Carbon Chloroform Extract (CCE) | GC - In House^c^ | mg L^-1^ | ND | 0.3 |
| Chloride (Cl) | APHA 4500-CI^-^ B | mg L^-1^ | 2 | 300 |
| Chloroform | GC - In House^d^ | mg L^-1^ | ND | 0.3 |
| Chromium (Cr) | APHA 3030 F / USEPA 6010 B | mg L^-1^ | ND (< 0.02) | 0.05 |
| Copper (Cu) | APHA 3030 F / USEPA 6010 B | mg L^-1^ | ND (< 0.02) | 2 |
| Cyanide (CN^-^) | APHA 4500-CN- E | mg L^-1^ | ND (< 0.02) | - |
| Fluoride (F^-^) | APHA 4500-F^-^ D | mg L^-1^ | 1.1 | 1.5 |
| Formaldehyde (CH_2_O) | APHA 6252 B | mg L^-1^ | < 0.1 | - |
| Free chloride residual (Cl_2_) | APHA 4500- Cl G | mg L^-1^ | < 0.1 | 5 |
| Hardness (CaCO_3_) | APHA 2340C | mg L^-1^ | < 1 | 500 |
| Iron (Fe) | APHA 3030 F / USEPA 6010 B | mg L^-1^ | 0.65 | 0.3 |
| Lead (Pb) | APHA 3030 F / USEPA 6010 B | mg L^-1^ | ND (< 0.02) | 0.01 |
| Magnesium (Mg) | APHA 3030 F / USEPA 6010 B | mg L^-1^ | 0.5 | - |
| Manganese (Mn) | APHA 3030 F / USEPA 6010 B | mg L^-1^ | ND (< 0.02) | 0.1 |
| Mercury (Hg) | APHA 3030 F / USEPA 6010 B | mg L^-1^ | ND (< 0.001) | 0.006 |
| Mineral oil | AOAC 945.102 (Mod.) | mg L^-1^ | ND (< 0.2) | - |
| Nickel (Ni) | APHA 3030 F / USEPA 6010 B | mg L^-1^ | ND (< 0.02) | 0.07 |
| Nitrate (NO_3_^-^) | APHA 4500-NO_3_ B | mg L^-1^ | < 0.1 | 50 |
| Nitrite (NO_2_^-^) | APHA 4500-NO_2_ B | mg L^-1^ | < 0.1 | 3 |
| Phenol (C_6_H_5_OH) | APHA 5530 D | mg L^-1^ | ND (< 0.002) | - |
| Phosphate (PO_4_^3-^) | APHA 3030 G / USEPA 6010 B | mg L^-1^ | 0.2 | - |
| Selenium (Se) | APHA 3030 F / USEPA 6010 B | mg L^-1^ | ND (< 0.005) | 0.04 |
| Silver (Ag) | APHA 3030 F / USEPA 6010 B | mg L^-1^ | ND (< 0.02) | - |
| Sodium (Na) | APHA 3030 F / USEPA 6010 B | mg L^-1^ | 27 | 50 |
| Sulfate (SO_4_) | APHA 4500-SO_4_ E | mg L^-1^ | 8 | 250 |
| Sulfur (S) | APHA 3030 F / USEPA 6010 B | mg L^-1^ | 3.9 | - |
| Total nitrogen | APHA 3030 F / USEPA 6010 B | mg L^-1^ | 5.6 | - |
| Zinc (Zn) | APHA 3030 F / USEPA 6010 B | mg L^-1^ | ND (< 0.02) | 5 |
| **Other** |  |  |  |  |
| Acidity | APHA 2310 B | mg L^-1^ | < 1 | - |
| Alkalinity | APHA 2320 B | mg L^-1^ | 76 | - |
| Biochemical oxygen demand (BOD) 5 days at 20 °C | APHA 5210 B | mg L^-1^ | 5 | - |
| Biochemical oxygen demand (BOD) 5 days at 60 °C | APHA 5210 B | mg L^-1^ | 10 | - |
| Biochemical oxygen demand (BOD) 5 days at 80 °C | APHA 5210 B | mg L^-1^ | 5 | - |
| Chemical oxygen demand (COD) | APHA 5220 B | mg L^-1^ | 35 | - |
| Total organic carbon (TOC) | APHA 5310 B | mg L^-1^ | 9.04 | - |
| **Bacteriological** |  |  |  |  |
| Total coliform count | APHA 9221 B | MPN  (per 100 mL) | ND < 1.1 | ND |
| *E. coli* | APHA 9221 E | MPN  (per 100 mL) | ND < 1.1 | ND |

Abbreviations: WHO, World Health Organization; APHA, in accordance with American Public Health Association, Standard Methods for the Examination of Water and Wastewater; USEPA, United States Environmental Protection Agency; GC, gas chromatography; AOAC, Association of Official Analytical Chemists; TCU, true color unit; NTU, Nephelometric Turbidity Unit; MPN, most probable number; Mod., modified method.

^a^ The biocide analysis includes screening of 2,4-dichlorophenoxyacetic acid, fluazifop, hexazinone, metsulfuron-methyl, 3-amino-1,2,4-triazole, glyphosate, and trichlorpy.

^b^ Biocides analysis was performed on an Agilent GC-MS instrument (Agilent Technologies, Palo Alto, CA, USA) according to NIST reference standards. Separation was achieved using a Phenomenex ZB-5 column.

^c^ Carbon chloroform extraction method involves the adsorption of organic matter on activated carbon. It then dried and extracted with chloroform using Soxhlet Extractor. Chloroform removal was performed by distillation prior weighing the extracted residue.

^d^ Chloroform was determined using Agilent DB-624 on headspace GC/MS (Agilent).
